# Single-Incision Laparoscopic Cholecystectomy in a Patient with a Left Ventricular Assist Device

**DOI:** 10.70352/scrj.cr.25-0756

**Published:** 2026-03-13

**Authors:** Ayako Ishii, Shohei Takaichi, Kei Fukumori, Watsapol Juavi Jitjan, Yuka Iwami, Kyoko Kobayashi, Satoshi Ishikawa, Masakatsu Paku, Kazuya Iwamoto, Tomofumi Ohashi, Yujiro Nakahara, Kohei Murakami, Hidekazu Takahashi, Tadafumi Asaoka, Ichiro Takemasa, Takeshi Omori

**Affiliations:** Department of Gastroenterological Surgery, Osaka International Medical and Science Center, Osaka Keisatsu Hospital, Osaka, Osaka, Japan

**Keywords:** single-incision laparoscopic cholecystectomy, left ventricular assist device, lower abdominal approach, driveline management, low-pressure pneumoperitoneum, anticoagulation management

## Abstract

**INTRODUCTION:**

Laparoscopic cholecystectomy (LC) is the standard treatment for benign gallbladder diseases, including acute cholecystitis (AC). However, performing LC in patients with a left ventricular assist device (LVAD) presents specific technical and physiological challenges related to driveline preservation and hemodynamic stability. We report the first case of single-incision laparoscopic cholecystectomy (SILC) via the right lower abdomen in a patient with an LVAD after treatment for biliary tract infection, following prior surgery for strangulated ileus and umbilical wound infection.

**CASE PRESENTATION:**

A 57-year-old male with dilated cardiomyopathy underwent LVAD implantation and later developed AC and cholangitis, managed with endoscopic retrograde biliary drainage. Three months later, he underwent laparoscopic adhesiolysis for a strangulated ileus via a midline incision, complicated by umbilical wound infection. After recovery, elective LC was planned. Preoperative fluoroscopy identified the driveline’s subcutaneous course through the upper midline and right upper abdomen. To avoid the infected area and driveline injury, SILC was performed via the right lower abdomen. Dense midline adhesions were manageable on the right side, and the driveline was clearly preserved. Pneumoperitoneum was maintained at 8 mmHg to minimize hemodynamic disturbance. Heparin was resumed 3 hours postoperatively and warfarin on POD 1. The patient was discharged on POD 7 without complications.

**CONCLUSIONS:**

This case demonstrates the feasibility of SILC via the right lower abdomen in an LVAD patient with a complex surgical history. A tailored surgical approach and multidisciplinary perioperative planning were essential for achieving a safe and effective surgery.

## Abbreviations

AC
: acute cholecystitis
LC
: laparoscopic cholecystectomy
LVAD
: left ventricular assist device
MAP
: mean arterial pressure
PI
: pulsatility index
PT-INR
: prothrombin time-international normalized ratio
SILC
: single-incision laparoscopic cholecystectomy

## INTRODUCTION

LC has become the standard treatment for benign gallbladder diseases, including AC.^1)^ An LVAD is a mechanical circulatory support system that helps maintain systemic perfusion in patients with advanced heart failure who are refractory to medical therapy.^2)^ With the expanding use of LVADs for advanced heart failure, the number of such patients requiring non-cardiac surgery continues to rise.^3)^ However, performing LC in patients supported by an LVAD presents specific technical and physiological challenges.^4,5)^ The driveline—a subcutaneous cable connecting the implanted pump to the external controller—represents a risk of injury during port placement and subsequent surgical manipulation, and pneumoperitoneum can alter preload and afterload, potentially compromising LVAD function.^6,7)^ Moreover, these patients are typically maintained on long-term anticoagulation, further complicating perioperative management.^8)^

Herein, we report the first case of SILC via the right lower abdomen in a patient with an LVAD, performed after treatment for biliary tract infection and previous surgery for strangulated ileus complicated by umbilical wound infection. This case highlights how a tailored surgical approach can achieve safe and effective minimally invasive surgery in patients with complex cardiologic and surgical backgrounds.

## CASE PRESENTATION

**Fig. 1:**
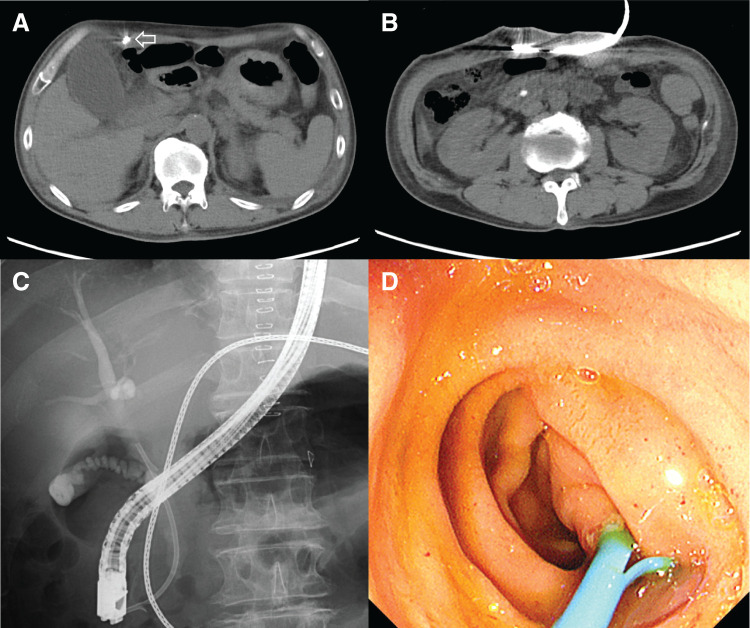
Preoperative imaging and endoscopic findings related to biliary tract infection. (**A**) Abdominal CT showed gallbladder wall thickening with a driveline running subcutaneously (arrow). (**B**) A stone is identified in the common bile duct. (**C**) Fluoroscopic image during endoscopic retrograde cholangiopancreatography confirmed placement of the ERBD tube. (**D**) Endoscopic image demonstrated placement of an ERBD tube through the papilla without sphincterotomy. ERBD, endoscopic retrograde biliary drainage

**Fig. 2:**
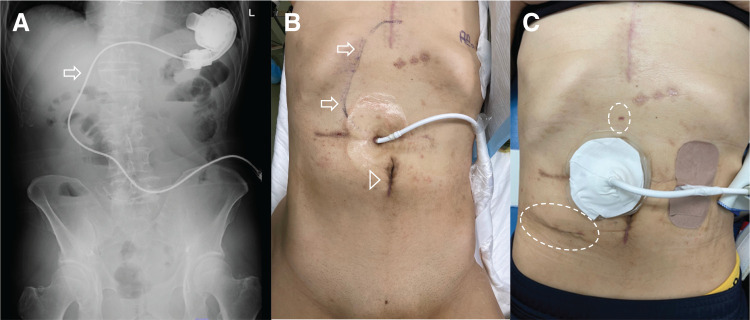
Pre- and postoperative findings related to the LVAD driveline and surgical access. (**A**) Preoperative fluoroscopic imaging shows the subcutaneous course of the LVAD driveline running from the epigastrium toward the right upper abdomen (arrow). (**B**) The driveline trajectory is marked on the abdominal skin (arrows) to prevent intraoperative injury; the arrowhead indicates the previous umbilical wound. (**C**) Postoperative clinical image shows the surgical wounds in the right lower abdomen and epigastrium (dotted circles). LVAD, left ventricular assist device

**Fig. 3:**
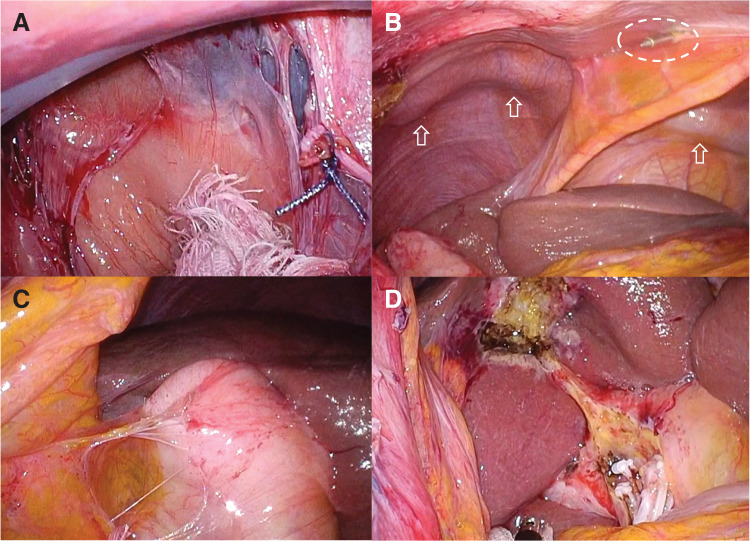
Intraoperative findings during SILC. (**A**) Dense adhesions were observed between the midline abdominal wall and the small intestine, while the right-sided adhesions were less severe and manageable. (**B**) The LVAD driveline was identified intraoperatively and carefully preserved throughout the procedure; the arrows indicate the driveline, and the dotted circle marks the 3-mm Endo Relief needle-forceps (Hope Denshi, Chiba, Japan). (**C**) Some adhesions were encountered around the gallbladder. (**D**) Cholecystectomy was successfully completed without conversion or the need for additional ports. LVAD, left ventricular assist device; SILC, single-incision laparoscopic cholecystectomy

A 57-year-old male underwent LVAD implantation at another hospital for the treatment of dilated cardiomyopathy. During his postoperative course, the patient developed AC and subsequent cholangitis. Abdominal CT showed gallbladder wall thickening with a driveline running subcutaneously (**Fig. 1A**) and a stone in the common bile duct (**Fig. 1B**). He was transferred to our hospital for further management. Endoscopic retrograde cholangiopancreatography was performed (**Fig. 1C**), and an endoscopic retrograde biliary drainage tube was placed without sphincterotomy due to ongoing anticoagulation (aspirin and warfarin) (**Fig. 1D**). Three months later, he developed strangulated ileus and was again transferred to our facility. Laparoscopic adhesiolysis for small bowel obstruction was performed via a midline incision. Postoperatively, he developed an umbilical wound infection requiring negative-pressure wound therapy. He was discharged 1 month later. Two months after discharge, elective LC was scheduled. Before surgery, we performed preoperative fluoroscopic imaging to identify the course of the driveline (**Fig. 2A**) and marked it on the abdominal skin to avoid intraoperative injury (**Fig. 2B**). The driveline was found to run subcutaneously through the upper midline and right upper abdomen, exiting cranially to the umbilicus. Given the previous abdominal surgery via a midline incision and wound infection, we chose a right lower abdominal approach for SILC. Perioperative anticoagulation management was carefully planned. Warfarin and aspirin were discontinued 1 week before surgery, and heparin bridging was initiated on the same day. Four days before surgery, laboratory tests confirmed good anticoagulation control, with an activated partial thromboplastin time of 47.5 s and a PT-INR of 1.23. For the SILC approach, a 3-cm incision was made in the right lower abdomen, and a Lap Protector Mini with EZ Access (three 5-mm ports) (Hakko, Nagano, Japan) was inserted. A 3-mm Endo Relief needle forceps (Hope Denshi, Chiba, Japan) was introduced with special care to avoid injuring the driveline running from the epigastrium (**Fig. 2C**). Pneumoperitoneum was established and maintained at an intra-abdominal pressure of 8 mmHg. LVAD-specific hemodynamic parameters were continuously monitored in collaboration with the cardiac anesthesia team, including pump flow, PI, and MAP. The average pump flow was 3.3 L/min before pneumoperitoneum, 3.2 L/min during pneumoperitoneum, and 3.4 L/min after desufflation. The average PI was 6.6 before pneumoperitoneum, 6.9 during pneumoperitoneum, and 5.8 after desufflation. The MAP was 71.9 mmHg before pneumoperitoneum, increased to 89.4 mmHg during pneumoperitoneum, and was 83.6 mmHg after desufflation. Intermittent administration of phenylephrine (0.1–0.2 mg boluses) was used as needed to maintain adequate arterial pressure. Throughout the procedure, pump flow, PI, and MAP remained within clinically acceptable ranges. Dense adhesions were found between the midline abdominal wall and small intestine, but the right-sided adhesions were less severe and manageable (**Fig. 3A**). The driveline was identified intraoperatively and preserved (**Fig. 3B**). Throughout the surgical procedure, the course of the driveline was continuously confirmed by direct visual identification and gentle palpation through the abdominal wall to ensure complete avoidance of driveline injury. Some adhesions were encountered around the gallbladder (**Fig. 3C**); however, cholecystectomy was successfully completed without conversion or the need for additional ports (**Fig. 3D**). Operative time was 210 min, including 25 min of careful hemostasis. Heparin administration was initiated 3 hours after surgery at a dose of 5000 units. With no signs of postoperative bleeding on laboratory data or vital signs, oral warfarin was resumed on POD 1, and the heparin dose was increased to 10000 units. On POD 3, the PT-INR was 1.35, and on POD 6, it increased to 1.74, showing a favorable trend toward therapeutic anticoagulation. In the absence of bleeding or thromboembolic events, and with close outpatient monitoring planned, the patient was discharged on POD 7. The patient is still on the waiting list for a heart transplantation.

## DISCUSSION

To our knowledge, this is the first case of SILC performed via a right lower abdominal approach in a patient with an LVAD. An LVAD is an implantable mechanical pump that supports systemic circulation by unloading the failing left ventricle and maintaining adequate end-organ perfusion in patients with advanced heart failure.^2)^ As the number of patients living with LVADs continues to rise, reflecting their expanding use as a bridge to transplantation, a bridge to candidacy, or destination therapy, non-cardiac surgeries in this population are becoming increasingly common.^9,10)^ LC in LVAD patients requires meticulous perioperative planning due to complex physiological and technical challenges, particularly the risk of injuring the subcutaneous driveline during trocar placement or dissection, which could result in device malfunction or infection and lead to life-threatening complications.^6)^ Previous reports have described LC in LVAD patients^4,5,11–25)^; however, no cases of SILC, including those using a lower abdominal approach, have been reported to date.

In the present case, the patient had a history of midline laparotomy for strangulated ileus and subsequent umbilical wound infection, rendering the standard umbilical approach inappropriate. Under these conditions, the single-incision laparoscopic approach itself offered several important advantages in this LVAD-supported patient. Compared with conventional multi-port laparoscopy, SILC allowed greater flexibility in selecting a single, well-controlled entry site away from both the driveline course and the previously infected umbilical region, thereby reducing the risk of inadvertent driveline injury. In addition, the use of a single incision minimized the number of fascial and skin punctures, which may be particularly beneficial in patients with implanted foreign material such as an LVAD driveline, where surgical site infection can have serious consequences. Furthermore, concentrating all instruments through a single access port facilitated continuous visual awareness of instrument trajectories, enabling clearer identification and preservation of the driveline throughout the procedure.

In this patient, the selection of the right lower abdominal access route was a key element of operative planning. At our institution, SILC has been established as the standard procedure for gallstone disease. Our experience, including 1469 cases reported by Furukawa et al., has demonstrated its safety and efficacy across a wide range of patients.^26)^ This experience provided a foundation for adapting the technique to this anatomically complex case, and alternative strategies were carefully considered. A standard umbilical SILC approach was contraindicated because of prior midline laparotomy and umbilical wound infection, and open surgery was considered more invasive with potentially higher cardiopulmonary risk. By contrast, the right lower abdominal approach allowed safe entry through less adherent tissue planes while maintaining distance from both the driveline course and the infected region. Chun et al. have also reported that, compared with the conventional umbilical approach, the lower abdominal approach offers comparable safety with superior cosmetic results.^27)^ Our successful application of this modified right lower abdominal SILC approach further supports its feasibility in LVAD patients with complex surgical histories.

LVAD patients are typically maintained on long-term anticoagulation, which increases perioperative bleeding risk, while interruption of therapy elevates the risk of thromboembolic or pump-related complications.^8)^ In this case, careful intraoperative hemostasis allowed for early, staged reinitiation of anticoagulation: intravenous heparin was resumed 3 hours postoperatively and transitioned to oral warfarin the following day. According to the International Society for Heart and Lung Transplantation consensus statement and the American Association for Thoracic Surgery guidelines, unfractionated heparin should be restarted as soon as adequate hemostasis is achieved—typically within 6–12 hours after surgery—to reduce the risk of pump thrombosis.^28)^ Thus, the anticoagulation strategy used here aligns with current expert recommendations and emphasizes the importance of close multidisciplinary management.

The establishment of pneumoperitoneum during LC may alter preload and afterload, potentially destabilizing circulatory dynamics in LVAD-supported patients.^7)^ In previous reports, intra-abdominal pressure during laparoscopy in LVAD patients was generally maintained at or above 10 mmHg.^4,5,12,14,15,17)^ On the other hand, the European Association for Endoscopic Surgery guidelines recommend using the lowest intra-abdominal pressure that allows adequate visualization of the surgical field to minimize adverse cardiopulmonary effects and postoperative pain.^29)^ Consistent with this principle, we maintained pneumoperitoneum at 8 mmHg, which contributed to stable hemodynamics throughout the procedure.

## CONCLUSIONS

This case demonstrates the importance of an individualized surgical strategy in LVAD patients undergoing LC, particularly when using a single-incision approach. By tailoring the operative approach based on anatomical constraints, prior surgical history, and infection control, safe and effective SILC was achieved. It further highlights the value of close collaboration among surgery, cardiology, and anesthesiology for multidisciplinary perioperative management, ensuring optimal anticoagulation and hemodynamic management to minimize perioperative complications. As LVAD implantation continues to expand, accumulating such procedural experience will contribute to refining surgical strategies and perioperative care for LVAD patients undergoing SILC or other laparoscopic procedures.

## DECLARATIONS

### Availability of data and materials

Data will be made available on reasonable request.
